# Cell Therapy for Refractory Angina: A Reappraisal

**DOI:** 10.1155/2017/5648690

**Published:** 2017-12-10

**Authors:** Beatrice Bassetti, Patrizia Nigro, Valentina Catto, Laura Cavallotti, Stefano Righetti, Felice Achilli, Paolo Scacciatella, Corrado Carbucicchio, Giulio Pompilio

**Affiliations:** ^1^Unità di Biologia Vascolare e Medicina Rigenerativa, Centro Cardiologico Monzino IRCCS, Via Carlo Parea 4, 20138 Milan, Italy; ^2^Cardiac Arrhythmia Research Centre, Centro Cardiologico Monzino IRCCS, Via Carlo Parea 4, 20138 Milan, Italy; ^3^Unità Operativa Cardiochirurgia, Centro Cardiologico Monzino IRCCS, Via Carlo Parea 4, 20138 Milan, Italy; ^4^Unità Operativa Cardiologia e UTIC, Azienda Ospedaliera San Gerardo, Via G. B. Pergolesi 33, 20052 Monza, Italy; ^5^Dipartimento Cardiovascolare e Toracico, Azienda Ospedaliera Universitaria Città della Salute e della Scienza, Corso Bramante 88, 10126 Turin, Italy; ^6^Dipartimento di Scienze Cliniche e di Comunità, Università degli Studi di Milano, Via Festa del Perdono 7, 20122 Milan, Italy

## Abstract

Cardiac cell-based therapy has emerged as a novel therapeutic option for patients dealing with untreatable refractory angina (RA). However, after more than a decade of controlled studies, no definitive consensus has been reached regarding clinical efficacy. Although positive results in terms of surrogate endpoints have been suggested by early and phase II clinical studies as well as by meta-analyses, the more recent reports lacked the provision of definitive response in terms of hard clinical endpoints. Regrettably, pivotal trials designed to conclusively determine the efficacy of cell-based therapeutics in such a challenging clinical condition are therefore still missing. Considering this, a comprehensive reappraisal of cardiac cell-based therapy role in RA seems warranted and timely, since a number of crucial cell- and patient-related aspects need to be systematically analysed. As an example, the large variability in efficacy endpoint selection appears to be a limiting factor for the advancement of cardiac cell-based therapy in the field. This review will provide an overview of the key elements that may have influenced the results of cell-based trials in the context of RA, focusing in particular on the understanding at which the extent of angina-related endpoints may predict cell-based therapeutic efficacy.

## 1. Introduction

Refractory angina (RA) is a chronic condition characterized by the presence of persistent angina or angina equivalents (≥3 months) caused by untreatable coronary artery disease (CAD) in patients with objective evidence of myocardial ischemia [1]. The presence of left ventricular (LV) dysfunction may worsen such a clinical scenario. Refractory angina patients are defined as being “no option” since they are deemed not amenable for conventional revascularization treatment for reasons related to diffuse coronary lesions, unsuitability to chronic total occlusion mechanical revascularization, or frailty from the presence of severe comorbidities. These pathological features may significantly affect the quality of life with regard to physical function and well-being as well as midterm fatality [2], although the most recent reports have documented a time-dependent increase in mortality rates (less than 4% at 1 year and 30% at 9 years) [3]. Outcome ameliorations have been ascribed to rigorous adoption of standard medical therapy, risk factor modification, and aggressive revascularization techniques [4]. Nevertheless, despite continuous advances in CAD management, particularly in the aged population, the number of patients suffering from RA and the correlated hospitalization costs are still growing [5]. In fact, available estimates suggest that RA affects between 600,000 and 1.8 million people in the USA with an incidence of 50,000 [5] and 30,000–50,000 new cases per year in the USA and continental Europe, respectively [6].

It is important to highlight that the medical care of these patients is particularly challenging. As a matter of fact, the therapeutic options and available directions from current guidelines are still limited [6]. Moreover, the interaction between clinical symptoms, myocardial perfusion abnormalities, coronary anatomy, and ventricular function are particularly complex in these individuals. Thus, novel treatments for RA management in patients nonresponsive to standard pharmacologic therapies and not amenable to mechanical revascularization procedures are evermore needed.

In this regard, recent efforts have been directed on the discovery of novel therapies based on innovative interventional techniques, neuromodulation, shockwave strategies, and cell-based interventions. In particular, in the analogy of other high-impact cardiac conditions as left ventricular dysfunction following ST-elevated acute myocardial infarction and chronic heart failure, cardiac cell-based therapy has emerged as a promising tool for the management of RA [7]. The application of cardiac cell-based therapy in this patient population was described for the first time in 2003 by Tse and coworkers [8]. After this preliminary experience, several uncontrolled and controlled phase I-II trials were conducted. Collectively, published studies have overtime demonstrated the safety and potential benefit of different cell types. In an attempt to shed light on the available results, 3 meta-analyses have specifically addressed cardiac cell-based therapy efficacy in RA [9–11]. Notably, the most recent work by Khan et al. [11], cumulatively taking into account 6 controlled trials with 365 enrolled patients, clearly showed that cardiac cell-based therapy has had a positive impact over standard of care in terms of myocardial perfusion, angina frequency, use of antianginal medications, change in the Canadian functional class, exercise tolerance, LV function, and major adverse cardiac events (MACE). Notably, 3 large rigorous clinical trials have been recently made available [12–14]. Unfortunately, the outcomes reported in these studies were not conclusive due to early discontinuation, for example, the regrettable decision on the behest of the study sponsor to prematurely stop the phase III RENEW trial which was sufficiently powered to definitively evaluate the safety and efficacy of intramyocardial injection of CD34^+^ cells in “no option” RA patients.

In the light of these and other inconclusive results [15, 16], a reappraisal of the therapeutic efficacy of cardiac cell-based therapy in RA seems imperative. In particular, a number of crucial aspects, including rigorous trial design, sample size, and endpoint assumptions, deserve to be carefully reconsidered. As an example, the huge discrepancy in the choice of endpoints probably represents the more relevant limiting factor for the lack of efficacy demonstration.

In this work, we have provided an updated overview of the results stemmed from available clinical trials focused on the role of cardiac cell-based therapy for the management of RA. Furthermore, we have comprehensively discussed ischemia-related outcomes, including objective and subjective endpoints, in order to facilitate a critical reappraisal of published studies on cardiac cell-based therapy for RA.

## 2. Overview of Cardiac Cell-Based Therapy Clinical Trials in RA

To date, twenty-six cell-based therapy trials have been conducted in the context of RA (see Table 1). Key aspects, including the number of patients treated, cell sources and types, baseline ejection fraction, delivery methods, and efficacy and safety outcomes, have been collected and analysed hereafter. As for the concomitance of LV dysfunction, when available, baseline EF below normal and/or the proportion of patients with LV impairment has been highlighted.

It is worth to mention that safety of cardiac cell-based therapy, defined as related serious adverse events (SAEs) and/or major adverse cardiac events (MACE) occurring during cell delivery, hospitalization, and follow-up, appears to be overall reassuring as for available studies.

### 2.1. Study Design

A significant proportion of published trials (*n* = 13) may be categorized as uncontrolled pilot or phase I studies that cumulatively enrolled 220 patients (21% of the entire accrual population), ranging from a minimum of 4 to a maximum of 38 subjects [8, 19–23, 26, 28, 29, 31, 33, 36, 39]. A total of 841 patients have been included in phase II randomized controlled trials (RCTs; *n* = 13) [12–17, 24, 30, 32, 34, 35, 40, 41]. The vast majority of these RCT reported the adoption of an appropriate placebo or saline group [13, 14, 17, 30, 34, 35, 38, 41, 42]. However, some other studies (for instance FOCUS-HF and PROGENITOR trials), in an attempt to overcome ethical and practical obstacles of a placebo group, employed alternative methodologies such as sham and mock injection procedures [15, 24]. To our knowledge, large confirmatory phase III studies are not yet available. The mean follow-up was 12 months (range: 3–36 months). Merely 23% of studies were able to prolong postoperative surveillance beyond 1 year.

### 2.2. Cell-Related Variables

**Figure 1:**
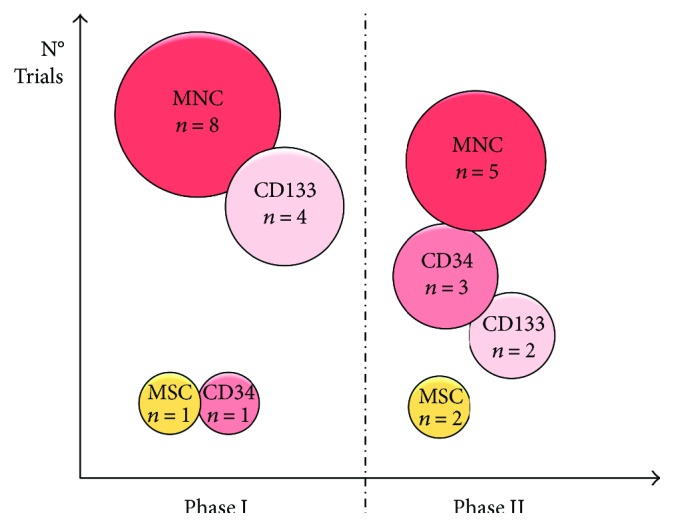
Cell types applied in clinical trials to treat RA. The figure depicts the number of published studies sorted for cell types and trial phases. MNC: mononuclear cells; MSC: mesenchymal stem cells; CD: cluster of differentiation.

Selected or unfractioned cells, primarily derived from the bone marrow (BM) [8, 13, 16, 19–24, 26, 28–33, 35, 36, 39–41] and to a lesser extent from peripheral blood (PB) [15, 17, 38] and, more recently, from the adipose tissue (AT), have been tested for their therapeutic potential in RA [14, 34]. Specifically, mononuclear cells (MNC), endothelial progenitor cells (such as CD34^+^ and CD133^+^ cells), and mesenchymal cells (MSC) have been subsequently investigated (Figure 1). It is worth highlighting that a direct comparison of different cell types is unfortunately not available. Notably, BM-derived MNC, the most extensively delivered cell type, have an unambiguously proven safety profile in the early and long-term period. Available data further suggested that BM-MNC therapy seemed effective in ameliorating myocardial perfusion and angina frequency [31, 42]. Moreover, the group of Mann et al. [31] showed that repeated BM cell injections in previously responding patients can reinforce clinical improvements. To date, such “first generation” cell lines have not been followed by more advanced “next generation” cell therapeutics. For instance, allogeneic applications, which have provided positive outcomes for other pathologic cardiac conditions [43–45], have not yet been explored in RA to date. Likewise, the so-called “second generation” stem cells, consisting of more purified cell populations, such as cardiac stem cells (CSC), cardiosphere-derived cells, or combination of cells (MSC/CSC or MSC/macrophage lineages), have not yet been examined in RA. Cumulatively, the big picture of on-going studies suggests that new early studies are still pursuing the path of autologous BM-MNC (Figure 1).

As for cell dose, unlike other cardiac settings, it has to be mentioned that there is a plethora of dose-escalation trials already available [14, 17–19, 29, 30, 35]. Most of these studies concerned PB-derived CD34^+^ cell dose finding, thanks to the effort of Losordo and coworkers [30]. Once verified in early safety of intramyocardial injection of 50,000 CD34^+^ cells/kg, in the subsequent phase IIb ACT34-CMI study, they compared the efficacy of two escalating dosages of CD34^+^ cells (1 × 10^5^ and 5 × 10^5^). Surprisingly, patients treated with the low dosage experienced greater improvements in angina frequency and exercise tolerance at 6 and 12 months, [17]. Nevertheless, after 24 months of follow-up, reduction in angina frequency was equivalent in both cell groups (*p* = 0.03) [18]. Additionally, Lee et al. [29] recently reported the superiority of 1 × 10^7^ cells with respect to 3 × 10^7^ CD34^+^ cells in improving heart function and clinical symptoms in patients with untreatable myocardial ischemia and LV dysfunction.

The dose-dependent response of BM-MNC has been further investigated in the PROTECT-CAD study [35] in which, however, no significant difference between the low- (1 × 10^6^ cells/0.1 mL) and the high-dose groups (2 × 10^6^ cells/0.1 mL) was observed in terms of total exercise time at 6 months of follow-up compared to baseline.

Therefore, based on currently available studies, there is no clear evidence of a dose-dependent effect of BM-derived cell delivery in RA patients. Further studies are thus necessary to completely address this question.

### 2.3. Cell Delivery

The injection technique is certainly a critical factor for the success and optimization of cell therapeutics. To date, a noticeable proportion of cardiac cell-based therapy trials in RA (*n* = 18) utilized advanced catheter-based endocavitary transendocardial cell administration strategy [8, 13–17, 21–26, 30–32, 34, 35, 38]. In this regard, the NOGA electromechanical mapping system has been proposed as an effective and reliable percutaneous method capable of discriminating between viable myocardium and scar tissue to precisely guide cell delivery into the target territories of the left ventricle. On the other hand, the fluoroscopy-guided strategy brings cost advantages and wider accessibility. In both cases, procedural safety has been consistently confirmed. Recently, in the context of the Phase I Trial of Endocavitary Injection of Bone Marrow Derived CD133^+^ Cells in Ischemic Refractory Cardiomyopathy (RECARDIO trial, NCT02059681), we have proposed a novel approach based on the integration of 3-dimensional (3-D) electroanatomical mapping (CARTO) with real-time intracardiac echocardiography (ICE) to guide the fluoroscopy-based infusion catheter [46].

Other delivery approaches included intracoronary [19, 29, 41] and intravenous infusion [39] and direct epicardial injection by open-chest surgery [20, 28, 33, 36]. Although no comparative studies are available to ascertain the most effective delivery option in RA, it appears that intramyocardial cell inoculation was by far the preferred delivery method in such a clinical context.

### 2.4. Efficacy Outcomes

**Figure 2:**
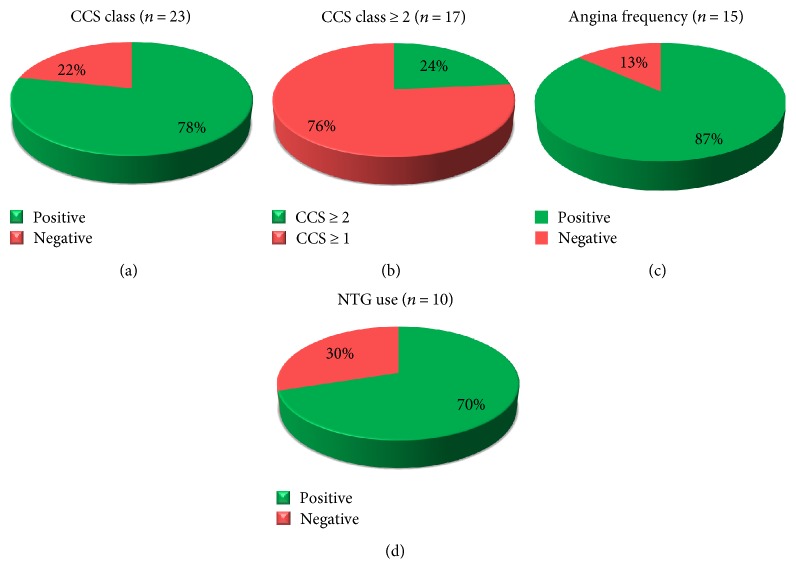
Indices of angina severity used to evaluate the effect of cardiac cell-based therapy in patients with RA. This schematic representation provides an analysis of the improvements observed in clinical trials relative to CCS class (a), CCS class greater than 2 (b), angina frequency (c), and nitroglycerine use (d). Positive and negative study outcomes have been defined according to statistical significance. The number of studies in the analysis is indicated between brackets. CCS: Canadian Cardiovascular Society; NTG: nitroglycerin.

**Figure 3:**
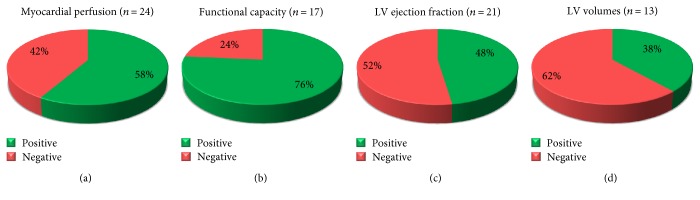
Indices of myocardial perfusion, functional capacity, and cardiac function used to evaluate the effect of cardiac cell-based therapy in patients with RA. Positive and negative study outcomes have been defined according to statistical significance. The number of studies in the analysis is indicated between brackets.

A wide spectrum of surrogate efficacy endpoints has been proposed to evaluate the efficacy of cell therapy in the RA setting, including indices of angina severity (Figure 2), as well as myocardial perfusion, exercise capacity, and cardiac function evaluations (Figure 3).

#### 2.4.1. Angina Assessment

As for the assessment of angina, the Canadian Cardiovascular Society (CCS) classification score (Figure 2(a)) was employed in the vast majority of available studies (*n* = 23). Notably, 78% of these trials reported a significant improvement in CCS angina class after cardiac cell-based therapy. Importantly, 24% of trials have described an improvement of CCS greater than 2 classes in cell-treated patients (Figure 2(b)). As an example, the randomized, placebo-controlled trial by Wang and coworkers [41] has described a remarkable reduction in CCS class from 3.3 ± 0.5 at baseline to 0.9 ± 1.0 at 6 months after intramyocardial delivery of CD34^+^ cells.

As an alternative evaluation, the number of anginal episodes per week as well as nitroglycerin (NTG) consumption has been proposed as valuable efficacy endpoints to describe changes in angina severity. Remarkably, in line with changes observed in CCS class, almost all studies reported ameliorations in terms of reduction in angina frequency and antianginal medications (Figures 2(c) and 2(d)). In this regard, the ACT34-CMI trial [17] suggested positive outcome benefits lasting over 2 years following intramyocardial administration of BM-derived CD34^+^ cells [18].

#### 2.4.2. Quality of Life and Health Status Evaluation

Patients' quality of life and health status have been investigated as pertinent variables strictly associated with angina severity. By the use of dedicated surveys, many studies documented a positive impact of cardiac cell-based therapy in terms of quality of life improvements. For instance, the FOCUS-HF trial adopted the Minnesota Living with Heart Failure and the Short Form-36 questionnaires to evaluate at 6 months over baseline the quality of life improvements in cell-treated and control patients [24]. In both questionnaires, patient scores improved only in the cell-treated group (*p* = 0.009 and *p* = 0.002, resp.). Other studies documented the changes in the quality of life using the disease-specific Seattle Angina Questionnaire (SAQ). Consistently, both Henry et al. [18] and van Ramshorst et al. [40] showed a significant increase in SAQ scores in these patients at 6 and 12 months.

#### 2.4.3. Myocardial Perfusion Assessment

Changes in regional myocardial perfusion were usually assessed by single-photon emission computed tomography (SPECT) imaging, with the aim to provide semiquantitative scores of the severity and extent of the perfusion defect. Specifically, changes in summed stress score (SSS), summed rest score (SRS), and summed difference score (SDS) were measured between the ends of follow-up and baseline. Remarkably, 24 out of 26 analysed trials were able to report a SPECT perfusion endpoint (Figure 3(a)). Overall, a significant percentage of studies (58%) suggested that the therapeutic delivery of cells leads to an objective improvement in myocardial perfusion. It is however worth mentioning that a considerable proportion of positive outcomes stemmed from uncontrolled phase I trials which enrolled a limited number of patients [8, 19–23, 31, 33, 36, 39]. Convincingly, positive data were also reported by more rigorous RCT [17, 40, 41], including the ACT34-CMI trial [17], which showed at 6 months of follow-up a significant improvement of the SSS in the cell group but not in controls (−117.4 ± 221.2 versus + 0.1 ± 161.1, *p* = 0.002). Conversely, disappointing findings arose from the recently published REGENT-VSEL [13] and ATHENA trials [14]. The latter randomized, double-blind study that enrolled in 1 : 1 ratio 31 patients versus controls failed to uncover statistical significant differences in SPECT parameters. Taken together, these findings suggest the need for an implementation and standardization of alternative methodologies, including positron emission computed tomography (PET) or cardiac magnetic resonance (cMR) imaging, to definitively document changes in regional and global myocardial perfusion at a microvascular level [17, 41].

#### 2.4.4. Functional Capacity Evaluation

As for functional capacity assessments, various objective endpoints were proposed, taking advantage of exercise testing with or without ventilatory gas exchange measurements. A variety of indicators was analysed, including exercise time, walking distance, maximal O_2_ consumption (MVO_2_), and other metabolic equivalents (METs). Cumulatively, such surrogate endpoints were reported to be positive in the majority of RCT, including the PROTECT-CAD [35], ACT34-CMI [17], ATHENA [14], and RENEW [12] trials (Figure 3(b)). In particular, the RENEW trial [38], the larger phase III study comparing intramyocardial delivery of CD34^+^ cells with respect to two control groups (placebo and standard care), was designed to detect changes in total exercise time (TET) at 12 months in 400 untreatable RA patients. Although the enrolment was prematurely stopped by the sponsor after the first 112 patients, study results, made recently available [12], showed the superiority of the cell therapy-treated patients compared with controls in improving TET (139 s ± 115 versus 69 s ± 122 s; *p* = 0.01). On the contrary, two consecutive reports by Perin et al. [16, 24] described the lack of MVO_2_ improvements in patients receiving BM cell therapy versus placebo, with the exception of a young patient subset (≤60 years). However, functional findings described here, when taken cumulatively, should be examined with caution since possible shortcomings, such as a wide heterogeneity of endpoints and the intrinsic limitations of such functional tests (e.g., subject compliance, orthopedic, or lower limb deficiencies), may have conditioned the outcomes.

Nevertheless, along with angina-related and perfusion endpoints, functional capacity assessments may play an important role in the context of future cardiac cell-based therapy studies in RA. Much effort should be given to the methodology for isolating improvements as well as for sample size selection. In this regard, from a methodological and regulatory standpoint, it is valuable to note that the mindful and weighted combination of the aforementioned indicators may offer a unique opportunity to provide efficacy evidence with relatively limited patient sample sizes.

#### 2.4.5. Global and Regional Cardiac Function Assessment

Cardiac cell-based therapy effects have been assessed by measuring global and regional cardiac function before and after cell administration. A variety of imaging methodologies with different acquisition and analysis protocols was applied. In the RA setting, left ventricular ejection fraction (LVEF) and volumes were assessed by cMR in 7 studies [8, 13, 28, 31, 34, 35, 40], by echocardiogram in 6 studies [14, 16, 22, 32, 33, 37], by SPECT in 4 studies [21, 23, 26, 39], and by combining different modalities in further 4 studies [15, 20, 24, 29]. In regard to LVEF changes, a significant number of studies (52%) revealed a lack of improvements at different follow-up time points (Figure 3(c)) [8, 13–15, 22–24, 31, 33, 34, 36]. In particular, when limiting the analysis to cMR evaluations, the majority of published studies were negative [8, 13, 28, 31, 34, 35, 40], with the exceptions of the PROTECT-CAD trial [35] that demonstrated at 6 months a significant improvement in global LVEF in BM-treated patients (*p* = 0.044) versus controls. Additionally, the study by van Ramshorst et al. [40] confirmed at 3 months an increase in LVEF in the cell-treated group only (change, 3%; 95% CI, 0.5% to 4.7% versus −1%; 95% CI, −2.1 to 1.1; *p* = 0.03).

In accordance with LVEF findings, a large proportion of studies (62%) also reported negative outcomes associated with LV volume measurements (Figure 3(d)).

Cumulatively, these data confirm the well-known assumption that the presence of a recovering ischemic (hibernating) myocardium after perfusion restoration is achievable in a minority of RA patients.

## 3. Future Perspectives

Is there a chance for cardiac cell-based therapy to be approved as a new therapy for *RA*? Overall, although extremely demanding, RA is undoubtedly, among cardiac conditions, the one in which cardiac cell-based therapy has shown very promising outcomes. Indeed, a consistent body of published trials cumulatively indicated that, although hard clinical endpoint has not been reached so far, cardiac cell-based therapy may increase the functional/perfusion status in a subset of RA patients as well as in a vast proportion of the quality of life, significantly reducing angina symptoms and antiangina medication usages.

Consistently, meta-analyses published to date showed that cardiac cell-based therapy has a clinically relevant impact over standard care [9–11]. Unfortunately and importantly, pivotal trials have failed to provide a definitive response so far, essentially due to strategically driven discontinuation of patient recruitment before reaching the prespecified sample size [12], low enrolment rates [13], or safety concerns [14]. Despite this, given the paucity of effective therapeutics, along with the extremely demanding clinical need, we consider cardiac cell-based therapy has a viable treatment option in RA for an addition to the therapeutic armamentarium of invasive cardiologists. It is worth noting that, using autologous BM-derived cells, intramyocardial cell delivery has been recently cleared for hospital-based reimbursement (Heart Centrum Leiden, Netherlands) as a percutaneous therapeutic strategy in the context of RA (code 979001088).

### 3.1. Novel Cell Types and Allogeneic Setting

Interestingly, alternative cell therapeutics and “off-the-shelf” settings are currently under investigation to overcome hurdles related to the lack of potency and cell impairments due to age and risk factors. For example, the “Safety and Efficacy of Human Umbilical-Cord-derived Mesenchymal Stem Cell Transplantation in Ischemic Cardiomyopathy” (UCMSC-Heart trial, NCT02439541) is currently recruiting patients with chronic heart ischemia and maximal tolerable angina to study the effect of the intracoronary infusion of MSC derived from umbilical cord blood (1 × 10^7^). In this study, the primary outcome is safety in terms of MACE in the first year after treatment while secondary outcomes are exercise time, perfusion at SPECT, LVEF measured by echocardiography, and clinical improvements. MSC are also studied in the “MESenchymal cell therapy And Myocardial Ischemia with decreased left ventricular function” program (MESAMI2 trial, NCT02462330). The major aim of this project is to evaluate the effect of intramyocardial administration of autologous BM-derived MSC (preconditioned with the pineal hormone melatonin) using the NOGA XP system in patients suffering from chronic ischemic cardiomyopathy with a NYHA class II-IV and/or angina CCS class III-IV.

## 4. Conclusions

In this review, we have provided a comprehensive outlook on the status of the cardiac cell-based therapy field in the RA context. Although available data are still inconclusive as for definitive efficacy, we acknowledge signals are present, as discussed in this review, that imply this strategy is the most valuable advanced treatment option, as a tool to ameliorate prognosis “*quad vitam and valitudinem*” in the therapeutic algorithm of patients suffering for RA.

## Figures and Tables

**Table 1:** Published cell therapy trials in RA.

Trial	Design	Treated/controls	Baseline LVEF%	Cell type	Delivery	FU (months)	Efficacy outcomes	Safety
ACT34-CMI [17, 18]	RCT, Phase II, dose finding	111/56	Normal	PB-CD34^+^	IM^endo^	6/12/24	↓ CCS and NTG use ↓ angina frequency in the low-dose group only ↑ perfusion ↑ exercise capacity in the low-dose group only	No differences, cell therapy versus control groups
Adler et al. [19]	Phase I, dose finding	9/0	Normal	BM-CD133^+^	IC	24	↔ angina frequency ↑ perfusion	No MACE reported
ATHENA ATHENA II [14]	RCT, phase II, dose finding	17/14	31.1 ± 8.2%	AT-MSC	IM^endo^	12	↓ CCS and NYHA ↔ perfusion ↑ MVO_2_ ↔ LVEF, LVEDV, and LVESV	No differences, cell therapy versus controls groups
Babin-Ebell et al. [20]	Phase I	6/0	66.67% of patients < 50%	BM-CD133^+^	IM^epi^	3/6/12	↓ CCS ↑ LVEF	1 possible cell related death
Beeres et al. [21]	Phase I	25/0	47% ± 13%	BM-MNC	IM^endo^	3/6/12	↓ CCS, angina frequency, and NTG use ↑ perfusion ↑ LVEF, LVESV, and RWM ↔ LVEDV	1 not cell therapy-related death and 1 MACE, 1 SAE
Briguori et al. [22]	Phase I	10/0	30% of patients < 50%	BM-MNC	IM^endo^	12	↓ CCS, angina frequency ↑ perfusion	1 not cell therapy-related MACE
Fuchs et al. [23]	Phase I	27/0	48 ± 9%	BM-MNC	IM^endo^	3/12	↓ CCS, angina frequency ↑ perfusion ↑ treadmill exercise ↔ LVEF	1 cell therapy-related periprocedural SAE (aortic dissection) 3 not cell therapy-related MACE at follow-up
FOCUS-HF [24]	RCT, phase II	20/10	37 ± 10.6% cell therapy group, 39 ± 9.1% control group	BM-MNC	IM^endo^	3/6	↓ CCS and ↔ NYHA ↑ perfusion ↔ MVO_2_ ↔ LVEF and LVWMI	SAEs evenly distributed between control and cell therapy groups
FOCUS-CCTRN [16]	RCT, phase II	61/31	34.7 ± 8.8% cell therapy group, 32.3 ± 8.6% control group	BM-MNC	IM^endo^	6	↔ CCS, NYHA and NTG use ↔ perfusion ↔ MVO_2_ ↑ LVEF ↔ LVESV, LVEDV, and LVWMI	No significant differences between control versus cell therapy groups
Haack-Sørensen et al. [25–27]	Phase I	31/0	Normal	BM-MSC	IM^endo^	12/36	↓ CCS, angina frequency, and NTG use ↔ perfusion ↑ MET ↑ LVEF	Not cell therapy-related SAEs and MACE
Klein et al. [28]	Phase I	10/0	15.8 ± 5	BM-CD133^+^	IM^epi^	9	↓ CCS ↑ LVEF, LVEDV	1 possible cell therapy-related death
Lee et al. [29]	Phase I, dose finding	38/0	Normal	PB-CD34^+^	IC	6/9/12	↓ CCS and NYHA ↔ perfusion ↑ LVEF, LVEDV, and LVESV	2 not cell therapy-related deaths 2 not cell therapy-related MACE
Losordo et al. [30]	RCT, phase II, dose finding	18/6	NA	PB-CD34^+^	IM^endo^	3/6/9/12	↓ CCS, angina frequency, NTG use ↔ perfusion ↑ exercise capacity	SAEs evenly distributed between control and cell therapy groups
Mann et al. [31]	Phase I	23/0	Normal	BM-MNC	IM^endo^	3/6/12	↓ CCS, angina frequency ↑ perfusion ↔ exercise capacity ↔ LVEF, LVEDV, and LVESV	No safety issues
Pokushalov et al. [32]	RCT, Phase II	55/54	27.8 ± 3.4% cell therapy group, 26.8 ± 3.8% control group	BM-MNC	IM^endo^	3/6/12	↓ CCS, NYHA, angina frequency and NTG use ↑ perfusion ↑ exercise capacity ↑ LVEF	Significant difference in mortality rate between cell therapy versus control groups (6 versus 21 deaths, resp.)
Pompilio et al. [33]	Phase I	5/0	40% of patients < 50%	BM-CD133^+^ PB-CD133^+^	IM^epi^		↓ CCS, angina frequency ↑ perfusion ↔ LVEF	No safety issues
PRECISE [34]	RCT, phase II	21/6	36.7 ± 7.5% cell therapy group, 34.2 ± 9.5% control group	AT-MSC	IM^endo^	6/12/18	↔ CCS and NYHA ↑ perfusion ↑ MVO2 and MET ↔ LVEF, LVEDV, and LVESV ↑ LV mass and LVWMI	SAEs evenly distributed between control and cell therapy groups
PROGENITOR [15]	RCT, phase II	19/9	Normal	PB-CD133^+^	IM^endo^	6/12/24	↔ CCS, angina frequency, and NTG use ↔ perfusion ↔ treadmill exercise ↔ LVEF, LVEDV, and LVESV	SAEs and MACE evenly distributed between control versus cell therapy groups
PROTECT-CAD [35]	RCT, phase II, dose finding	19/9	51.9 ± 8.5% cell therapy group, 45.7 ± 8.3% control group	BM-MNC	IM^endo^	3/6	↔ CCS ↓ NYHA ↔ perfusion ↑ treadmill exercise ↑ LVEF ↔ LVEDV and LVESV	1 death and 1 MACE in control group 1 SAE in cell therapy group at follow-up
ReACT [36, 37]	Phase I	14/0	Normal	BM-MNC	IM^epi^	3/6/12	↓ CCS ↑ perfusion ↔ LVEF	2 deaths (1 for cardiogenic shock due to MI, 1 MI)
RegentVsel [13]	RCT, phase II	16/15	Normal	BM-CD133^+^	IM^endo^	4/12	↔ CCS and NTG use ↔ perfusion ↔ LVEF ↑ LV volumes	MACE evenly distributed between control versus cell therapy groups 1 SAE in cell therapy group (pseudoaneurysm of the femoral artery)
RENEW [12, 38]	RCT, phase II	57/55	Normal	PB-CD34^+^	IM^endo^	3/6/12	↓ angina frequency ↑ exercise capacity	No safety issues
Tse et al. [8]	Phase I	8/0	Normal	BM-MNC	IM^endo^	3	↓ angina frequency and NTG ↑ perfusion ↔ LVEF ↑ target wall thickening and motion	No MACE reported
Tuma et al. [39]	Phase I	14/0	31.2%	BM-MNC	IV	24	↓ CCS, angina frequency ↑ perfusion ↑ exercise capacity ↑ LVEF	No safety issues
van Ramshorst et al. [40]	RCT, phase II	25/25	Normal	BM-MNC	IM^endo^	3/6	↓ CCS ↑ perfusion ↑ LVEF ↑ exercise capacity ↔ LVEDV and LVESV	MACE and SAEs evenly distributed between control versus cell therapy groups
Wang et al. [41]	RCT, phase II	56/56	NA	BM-CD34^+^	IC	3/6	↓ CCS, angina frequency, NTG use ↑ perfusion ↑ exercise capacity	No significant differences between control versus cell therapy groups

AT: adipose tissue; BM: bone marrow; CABG: coronary artery bypass grafting; FU: follow-up; LVEDV: left ventricular end-diastolic volume; LVEF: left ventricular ejection fraction (normal EF defined as ≥50%); LVESV: left ventricular end-systolic volume; LVWMI: left ventricular wall motion index; MACE: major adverse cardiac events; MET: maximal metabolic equivalent; MI: myocardial infarction; MNC: mononuclear cells; MSC: mesenchymal cells; NA: not available; NTG: nitroglycerin; PB: peripheral blood; PCI: percutaneous coronary intervention; RCT: randomized controlled trial; RWM: regional wall motion; SAEs: serious adverse events.

## Abbreviations

AT: Adipose tissue
BM: Bone marrow
CAD: Coronary artery disease
CCS: Canadian Cardiovascular Society
cMR: Cardiac magnetic resonance
CSC: Cardiac stem cells
LV: Left ventricular
LVEF: Left ventricular ejection fraction
MACE: Major adverse cardiac events
MET: Metabolic equivalent
MNC: Mononuclear cells
MSC: Mesenchymal stromal cells
MVO_2_: Maximal O_2_ consumption
NTG: Nitroglycerin
PB: Peripheral blood
RA: Refractory angina
RCT: Randomized controlled trial
RWM: Regional wall motion
SAE: Serious adverse event
SAQ: Seattle Angina Questionnaire
SPECT: Single-photon emission computed tomography
SSS: Summed stress score
SRS: Summed rest score
SDS: Summed difference score
TET: Total exercise time.
